# Vancomycin and High Level Aminoglycoside Resistance in* Enterococcus* spp. in a Tertiary Health Care Centre: A Therapeutic Concern

**DOI:** 10.1155/2016/8262561

**Published:** 2016-03-07

**Authors:** Seema Mittal, Pooja Singla, Antariksha Deep, Kiran Bala, Rama Sikka, Meenu Garg, Uma Chaudhary

**Affiliations:** ^1^BPS GMC (Women), Khanpur Kalan, Sonepat, Haryana, India; ^2^SHKMC, Mewat, Haryana, India; ^3^PGIMS, Rohtak, Haryana, India

## Abstract

*Aims*. This study was aimed at knowing the prevalence of vancomycin and high level aminoglycoside resistance in enterococcal strains among clinical samples.* Study Design*. It was an investigational study.* Place and Duration of Study*. It was conducted on 100* Enterococcus* isolates, in the Department of Microbiology, Pt. BDS PGIMS, Rohtak, over a period of six months from July to December 2014.* Methodology*. Clinical specimens including urine, pus, blood, semen, vaginal swab, and throat swab were processed and* Enterococcus* isolates were identified by standard protocols. Antibiotic sensitivity testing of enterococci was performed using Kirby-Bauer disc diffusion method.* Results*. High level gentamicin resistance (HLGR) was more common in urine samples (41.5%) followed by blood (36%) samples. High level streptomycin resistance (HLSR) was more common in pus samples (52.6%) followed by blood samples (36%). Resistance to vancomycin was maximum in blood isolates.* Conclusion*. Enterococci resistant to multiple antimicrobial agents have been recognized. Thus, it is crucial for laboratories to provide accurate antimicrobial resistance patterns for enterococci so that effective therapy and infection control measures can be initiated.

## 1. Introduction

*Enterococcus* has emerged as a nosocomial pathogen in the last two decades, causing urinary tract infections, genital tract infections, and endocarditis due to its colonizing capacity and multidrug resistance [1, 2]. The emergence of multidrug resistant enterococci to commonly used antibiotics, for example, aminoglycosides and cephalosporins, is because of their ability to attain and transfer the drug resistance gene, giving rise to enterococci with high level aminoglycoside (HLAR) and glycopeptide resistance [3]. They are less susceptible to penicillins and aminoglycoside antibiotics. However, combinations of penicillins with aminoglycosides are synergistically bactericidal against enterococci in vitro and are effective in treating severe enterococcal endocarditis. The mechanism of this synergy has been explained by the enhanced uptake of aminoglycosides in the presence of penicillins or other agents which inhibit cell wall synthesis [4]. An increased frequency of high level resistance to aminoglycoside antibiotics (MIC > 8,000 *μ*g/mL) in clinical isolates of enterococci has been reported which were also resistant to synergism with the penicillins [5].

This study was aimed at determining the antibiotic susceptibility of enterococci isolated from various clinical samples with reference to aminoglycoside and vancomycin. So if the knowledge of HLAR prevalence is available, clinicians can prescribe the various drug combination (cell wall inhibitor + aminoglycosides) at the very beginning of treatment avoiding the unnecessary usage of other antimicrobials.

## 2. Material and Methods

This study was conducted on 100 isolates of* Enterococcus* spp. from various clinical specimens including urine, pus, blood, semen, vaginal swab, and throat swab during a period of six months from July to December 2014. The samples were processed immediately after collection and* Enterococcus* isolates were identified by standard protocols based on Gram's staining, colony morphology, catalase test, bile solubility, growth in sodium chloride, bile esculin test, and sugar fermentation tests [6].

Antibiotic sensitivity testing of enterococci was performed using Kirby-Bauer disc diffusion method and Mueller-Hinton agar supplemented with 5% sheep blood was used as per CLSI guidelines [7]. The antibiotics discs used were penicillin (10 U), gentamicin (10 *μ*g), ciprofloxacin (5 *μ*g), linezolid (30 *μ*g), vancomycin (30 *μ*g), erythromycin (15 *μ*g), and doxycycline (30 *μ*g), and nitrofurantoin (300 *μ*g) was also added in urinary isolates.

HLAR in enterococci was detected by disc diffusion method. In disc diffusion method, high level (120 *μ*g) gentamicin and streptomycin (300 *μ*g) discs were placed on the agar medium. Plates were incubated at 37°C for 24 hours, and diameter of zone of inhibition was measured. Resistance was indicated by no zone and susceptibility by a zone of diameter ≥10 mm. The test was quality controlled using* E. faecalis* ATCC 29212 (susceptible) and* E. faecalis* ATCC 51299 (resistant) [8].

Vancomycin resistance was determined by using E-strip (Hi-media) on blood agar for those isolates which were resistant by disc diffusion test.

## 3. Results

Out of a total of 100 isolates, 41% from urine and semen and 25% from blood followed by 19% from pus and 8% from body fluid drains were included in the study. The majority of clinical samples from which* Enterococcus* spp. were recovered were from indoor (60%) in comparison to outdoor (40%) patients.

*Enterococcus* isolates from blood samples were resistant to penicillin (64%), and urinary isolates were resistant to fluoroquinolones (53.6%). Commonly used antimicrobials were found to be sensitive in* Enterococcus* spp. recovered from vaginal swab samples (Table 1).

High level gentamicin resistance (HLGR) was more common in urine samples (41.5%) followed by blood (36%) samples. High level streptomycin resistance (HLSR) was more common in pus samples (52.6%) followed by blood samples (36%) (Table 2).

In Table 3, more resistance to nitrofurantoin in* Enterococcus* isolates from urine samples was not noticed in indoor (17%) patients in comparison to OPD (7.3%). Similarly resistant to glycopeptides (vancomycin), fluoroquinolones were more common in hospitalized patients. Also, HLAR was more common in indoor (39%) versus outdoor patients (25%) (Table 3).

Resistance to vancomycin was maximum in blood isolates, that is, 32%. It was more common in IPD patients (28%) as compared to OPD patients (4%). High level aminoglycoside resistance was more common in vancomycin resistant enterococci (VRE) isolates than vancomycin sensitive enterococci (VSE) isolates. Five out of nine of the* Enterococcus* isolates resistant to vancomycin by disk diffusion test were found resistant to these glycopeptides by E-strip test (Table 4).

## 4. Discussion

Over the last few years, they have become important nosocomial pathogens probably due to inherent resistance to antibiotics (cephalosporins), ability to adhere to indwelling medical devices, and ability to survive in adverse environmental conditions. Antimicrobial resistance in* Enterococcus* has been increasing mainly in hospitalized patients [9–11].

In our study, out of the total 100* Enterococcus* isolates from different clinical specimens, the majority were recovered from hospitalized (60%) patients in comparison to outdoor (40%) patients, similar to other studies [12].

A combination of penicillin and aminoglycosides is considered as treatment of choice for enterococcal infections; therefore, resistance against these antibiotics has important clinical results effecting therapeutic prognosis. In the present study,* Enterococcus* isolates from blood samples were found to be penicillin resistant in 64% strains (16/25 = 64%) (MIC ranges from 16 to 32 *μ*g/mL), which could be due to resistance mechanism involving low affinity penicillin binding proteins or production of *β* lactamases.

In this study, prevalence of drug resistance to various antibiotics was as follows: ciprofloxacin (25%), penicillin (66.67%), and nitrofurantoin in urinary isolates (24.3%). Linezolid and vancomycin were found sensitive in 99% and 95% isolates of enterococcus. One isolate of* Enterococcus* spp. was found to be resistant to linezolid having inhibitory zone of diameter less than 15 mm. This type of antibiogram has been documented in earlier studies also [12–14].

Here a low prevalence of fluoroquinolone (25%) and other antibiotic resistance was found in comparison to other studies, 72% [12] and 62% [13], respectively, which could be due to very precise and judicious use of this antimicrobial in our institute.

The present study demonstrated high prevalence of HLAR (gentamicin and streptomycin) 29% and 35%, respectively. HLGR was more common in urine samples (41.5%) followed by blood (36%) samples. These findings are also reported in some studies [15]. However, a higher and lower prevalence level of HLGR and HLSR have been reported in few studies, respectively [13, 14, 16].

In our study, HLAR to both gentamicin and streptomycin was found in 22% isolates, specifically more in blood isolates. Some studies reported higher prevalence of HLAR to both gentamicin and streptomycin [16].

HLAR in these enterococcal strains nullify the efficacy of beta lactam and aminoglycoside combination therapy. Therefore, differentiation of HLAR from simple intrinsic resistance is important and should be adopted as a part of routine microbiology laboratories.

In this study, it was found that HLAR was more common in IPD (28%) as compared to OPD patients (4%), similar to other studies [12].

Vancomycin resistance was found in nine isolates of* Enterococcus* by disc diffusion method; out of nine isolates five isolates were confirmed as VRE on E-strip test having MIC (>64 *μ*gm/mL).

In India, the prevalence of VRE has been reported to be between 0 and 30 per cent [17–22]. In the present study, resistance to vancomycin was maximum in blood isolates, that is, 16.25% (4/25), with more prevalence in indoor patients (Table 4). It could be explained by the facts that in hospitalized patients use of broad spectrum antimicrobials is common practice and it leads to colonization pressure for selection of vancomycin resistance strains [23]. It may increase the risk of cross-infection among hospitalized patients via staff members and environmental contamination with VRE strains.

Out of four VRE strains, three were found to be sensitive to higher concentration of either of or both the aminoglycosides (Table 5). So the combination of the higher level aminoglycoside with cell wall inhibitor can be considered for treatment of VRE infection after antibiotic susceptibility testing.

*Control Efforts*. Due to lack of effective therapy for multiple antibiotic resistant enterococcal infection, prevention of the dissemination of these strains is of paramount significance. A very precise use of antimicrobials, for example, cephalosporins [24], and anti aerobic drugs [25] should be in practice. There are certain recommendations to reduce the cross-contamination by these organisms which include surveillance for colonization, identification of colonized and infected patients with their isolation, the use of gowns and gloves by health staff (barrier method), hand washing with an antiseptic after gloves removal, and avoiding contact with environmental surfaces after gloves removal. Medical equipment, for example, stethoscopes and blood pressure cuffs, must be dedicated to HLR patients. Environmental decontamination is also required with effective disinfectants (isopropyl alcohol, hypochlorite, and phenolic and quaternary ammonium salts) [26–29].

## 5. Conclusion

Deficiency of effective antimicrobial therapy and control measure for prevention of dissemination for multiple drug resistant enterococci are among the major factors for increasing prevalence of VRE and HLAR. Such strains pose therapeutic failure for clinicians. Thus, it becomes important for laboratories to provide accurate antimicrobial resistance patterns for enterococci so that effective therapy and infection control measures can be initiated. It becomes equivalently important that clinicians who are in direct contact with patients should go primarily for first/low generation of antibiotics for simple infections, for example, sore throat, rather than switching to higher class of antimicrobials.

## Figures and Tables

**Table 1:** Antibiotic resistance pattern of *Enterococcus *spp. in clinical specimen by disc diffusion test.

	Urine/semen, *n* = 41 (%)	Pus, *n* = 19 (%)	Blood, *n* = 25 (%)	High vaginal swab, *n* = 7 (%)	Drain, *n* = 8 (%)
Nitrofurantoin	10 (24.4%)	—	—	—	—
Linezolid	0	0	0	0	1 (12.5%)
Vancomycin	1 (2.4%)	0	4 (16.25%)	0	0
Penicillin	—	—	16 (64%)	—	4 (50%)

Erythromycin	20 (49%)	8 (42%)	1 (4%)	0	1 (12.5%)
Doxycycline	12 (29%)	0	0	0	0
Ciprofloxacin	22 (53.6%)	3 (15.7%)	0	0	0

**Table 2:** Prevalence of High level resistance in *Enterococcus *spp. isolates.

Antibiotic	U/S *n* = 41 (%)	Pus, *n* = 19 (%)	Blood, *n* = 25 (%)	HVS, *n* = 7 (%)	Drain, *n* = 8 (%)
Gentamicin (120 *μ*g)	17 (41.5%)	3 (16%)	9 (36%)	0	0
Streptomycin (300 *μ*g)	14 (34%)	10 (52.6%)	9 (36%)	1 (14%)	1 (12.5%)

U/S: urine/semen; HVS: high vaginal swab.

**Table 3:** Prevalence of antibiotic resistance in *Enterococcus* spp. in various clinical samples in IPD/OPD settings.

	U/S, *n* = 41		Pus, *n* = 19		Blood, *n* = 25		HVS, *n* = 7		Drain, *n* = 8	
	IPD	OPD	IPD	OPD	IPD	OPD	IPD	OPD	IPD	OPD
Nitrofurantoin	7 (17%)	3 (7.3%)	—	—	—	—	—	—	—	—
Linezolid	0	0	0	0	—	—	0	0	1 (12.5%)	0
Vancomycin	1 (2.4%)	0	0	0	3 (12%)	1 (4%)	0	0	0	0
Penicillin	—	—	—	—	12 (48%)	4 (16%)	—	—	4 (50%)	0

Erythromycin			6 (31.5%)	2 (10.5%)	1 (4%)	0	0	0	1 (12.5%)	0

Doxycycline	5 (12%)	7 (17%)	0	0	0	0	0	0	0	0
Ciprofloxacin	12 (29%)	10 (24%)	3 (15.7%)	0	0	0	0	0	0	0
HLG	7 (17%)	10 (24%)	3 (15.7%)	0	7 (28%)	2 (8%)	0	0	0	0
HLS	5 (12%)	9 (22%)	9 (47%)	1 (5%)	7 (28%)	2 (8%)	0	1 (14.3%)	1 (1.25%)	0

U/S: urine semen; HVS: high vaginal swab.

**Table 4:** Pattern of vancomycin susceptibility in *Enterococcus* spp. in various clinical specimens.

Clinical specimen	Vancomycin susceptible *n* (%)	Vancomycin resistant *n* (%)
Urine/semen	40	1
Pus	19	0
Blood	21	4
High vaginal swab	7	0
Drain fluid	8	0
Total	95	5

**Table 5:** Prevalence of HLAR in VRE.

VRE in clinical samples	HLGR	HLSR	HLGR + HLSR
Urine (*n* = 1)	1 (100%)	0	0
Blood (*n* = 4)	3 (75%)	3 (75%)	3 (75%)
